# ASFV-G-∆I177L as an Effective Oral Nasal Vaccine against the Eurasia Strain of Africa Swine Fever

**DOI:** 10.3390/v13050765

**Published:** 2021-04-27

**Authors:** Manuel V. Borca, Elizabeth Ramirez-Medina, Ediane Silva, Elizabeth Vuono, Ayushi Rai, Sarah Pruitt, Nallely Espinoza, Lauro Velazquez-Salinas, Cyril G. Gay, Douglas P. Gladue

**Affiliations:** 1 Plum Island Animal Disease Center, ARS, USDA, Greenport, NY 11944, USA; Elizabeth.Ramirez@usda.gov (E.R.-M.); Ediane.Silva@usda.gov (E.S.); Elizabeth.Vuono@usda.gov (E.V.); ayushi.rai@usda.gov (A.R.); Sarah.Pruitt@usda.gov (S.P.); Nallely.Espinoza@usda.gov (N.E.); Lauro.Velazquez@usda.gov (L.V.-S.); 2 Department of Anatomy and Physiology, Kansas State University, Manhattan, KS 66506, USA; 3 Department of Pathobiology and Population Medicine, Mississippi State University, P.O. Box 6100, Starkville, MS 39762, USA; 4 Oak Ridge Institute for Science and Education (ORISE), Oak Ridge, TN 37830, USA; 5 Agricultural Research Service, U.S. Department of Agriculture, Beltsville, MD 20705, USA; Cyril.Gay@usda.gov

**Keywords:** ASFV, I177L, African swine fever, ASF, African swine fever virus, vaccine, swine

## Abstract

The African swine fever virus (ASFV) is currently causing a pandemic affecting wild and domestic swine from Western Europe to Asia. No commercial vaccines are available to prevent African swine fever (ASF), resulting in overwhelming economic losses to the swine industry. We recently developed a recombinant vaccine candidate, ASFVG-ΔI177L, by deleting the I177L gene from the genome of the highly virulent ASFV strain Georgia (ASFV-G). ASFV-G-ΔI177L has been proven safe and highly efficacious in challenge studies using parental ASFV-G. Here, we present data demonstrating that ASFV-G-ΔI177L can be administered by the oronasal (ON) route to achieve a similar efficacy to that of intramuscular (IM) administration. Animals receiving ON ASFV-G-ΔI177L were completely protected against virulent ASFV-G challenge. As previously described, similar results were obtained when ASFV-G-ΔI177L was given intramuscularly. Interestingly, viremias induced in animals inoculated oronasally were lower than those measured in IM-inoculated animals. ASFV-specific antibody responses, mediated by IgG1, IgG2 and IgM, do not differ in animals inoculated by the ON route from that had IM inoculations. Therefore, the ASFV-G-ΔI177L vaccine candidate can be administered oronasally, a critical attribute for potential vaccination of wild swine populations.

## 1. Introduction

African swine fever virus (ASFV) is the causative agent of African swine fever (ASF), a devastating disease affecting domestic and wild pigs. ASF outbreaks are currently affecting Central and Eastern Europe and Asia, causing significant economic losses on a global scale [1]. ASFV is a structurally complex enveloped virus containing a large (180–190 kilobase pairs) double-stranded DNA genome encoding for over 150 open reading frames (ORFs) [1]. Dissemination and maintenance of the virus in susceptible wild swine populations makes management of a disease outbreak difficult. Because no commercial vaccine is available, outbreak management relies on restricting animal movements and culling infected herds [1]. Live attenuated viruses, developed by genetic manipulation of virulent strains, have been shown experimentally to be effective vaccines [2,3,4,5,6,7,8,9,10]. Most of these experimental vaccines are administered by the parenteral route. Administering ASFV vaccines by the oral route would allow for their use in wild animals, facilitating the regional control or eradication of the disease.

Recently we reported the rational development of a live attenuated vaccine candidate, ASFV-G-ΔI177L, obtained by deletion of the I177L gene from the genome of ASFV-G [6]. ASFV-G-ΔI177L was shown to be safe when parenterally inoculated at high doses and highly efficacious in inducing protection against challenge with the parental, highly virulent ASFV-G when administered in a relatively low dose. 

Here, we demonstrate that oronasal (ON) administration of ASFV-G-ΔI177L achieves similar efficacy as an intramuscular (IM) injection. ON-inoculated animals were protected against challenge with the virulent parental ASFV-G. Efficacy of protection as well as virus-specific antibody responses do not differ in animals inoculated with ASFV-G-ΔI177L by either the ON or IM route

## 2. Materials and Methods

### 2.1. Cell Culture and Viruses

Primary cultures of swine macrophages were prepared from swine blood, following procedures previously described. Preparation of macrophage cultures in 96-well plates for virus titration was also performed as previously described [11].

Development of the live attenuated vaccine candidate strain ASFV-G-ΔI177L has been previously described [6]. Briefly, partial deletion of the I177L gene in the genome of highly virulent isolate Georgia (ASFV-G) was replaced by mCherry under the ASFV p72 promoter. 

Virus titration was performed on primary swine macrophage cell cultures in 96-well plates. Virus dilutions and cultures were performed using macrophage medium. The presence of virus was assessed by hemadsorption (HA) [6], and virus titers were calculated by the Reed and Muench method [12].

ASFV-G used in the animal challenge experiments is a field isolate kindly provided by Nino Vepkhvadze from the Laboratory of the Ministry of Agriculture (LMA) in Tbilisi, Republic of Georgia.

### 2.2. Animal Experiments

All animal experiments were done in a BSL-3ag facility at Plum Island Animal Disease Center (Greenport, NY, USA). Protective efficacy of ASFV-G-∆I177L was assessed using 80- to 90-pound commercial breed swine. Groups of crossbreed Yorkshire pigs (*n* = 5) were inoculated either intramuscularly with 10^2^ HAD_50_ or oronasally with 2 × 10^6^ HAD_50_ (10^6^ HAD_50_ instilled in the rear of the nasal cavity and 10^6^ HAD_50_ at the base of the tongue) of ASFV-G-∆I177L. A sixth animal (sentinel) was not inoculated and cohabitated for 28 days with the inoculated animals. Sentinel animals were removed at the time of challenge. A mock-inoculated group (*n* = 5) was included as a control. Clinical signs (anorexia, depression, fever, purple skin discoloration, staggering gait, diarrhea, and cough) and changes in body temperature were recorded daily throughout the experiment. Animals inoculated with ASFV-G-ΔI177L were given an IM challenge 28 days later with 10^2^ HAD_50_ of the parental virulent ASFV-G strain. Clinical signs associated with the disease were recorded as described earlier [6].

### 2.3. Detection of Anti-ASFV Antibodies

The presence of virus-specific antibodies in the sera of the inoculated pigs was evaluated using an in-house indirect ELISA, which is described elsewhere [13]. The virus antigen was produced from Vero cells infected with a Vero-adapted ASFV strain. ELISA plates (MaxiSorp, Nunc, St. Louis, MO, USA) were coated with infected or uninfected cell extract (1 μg per well). The plates were blocked with 10% skim milk (Merck, Kenilworth, NJ, USA) in phosphate-buffered saline and 5% normal goat serum (Sigma, St. Louis, MO, USA). Each swine serum was tested at multiple dilutions against both infected and uninfected cell antigens. ASFV-specific antibodies in the swine sera were detected by an anti-swine IgM-, IgG-, IgG1- or IgG2-horseradish peroxidase conjugate (KPL, Gaithersburg, MD, USA) and SureBlue Reserve peroxidase substrate (KPL). Plates were read at an optical density of 630 nm (OD_630_) in an ELx808 plate reader (BioTek, Shoreline, WA, USA). Serum titers were expressed as the log_10_ of the highest dilution, where the OD_630_ reading of the tested sera at least duplicates the reading of the mock-infected sera.

## 3. Results

### 3.1. Replication of ASFV-G-ΔI177L in Animals Inoculated by Either IM or ON Route

**Figure 1:**
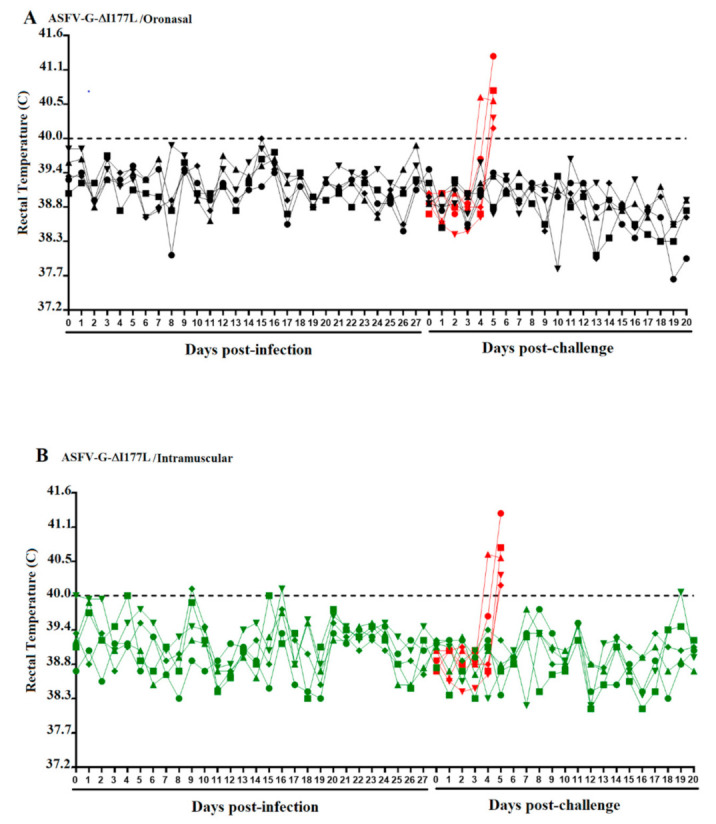
Kinetics of body temperature values in pigs oronasally (**A**) or intramuscularly (**B**) inoculated with ASFV-G-ΔI177L before (Days post-infection) and after challenge (Days post-challenge) with ASFV-G. Each curve represents individual data from each of the animals under each of the treatments. Red curves represent a group of mock-inoculated animals used as a control during both challenge experiments.

We first evaluated the ability of ASFV-G-ΔI177L to replicate systemically in swine after ON administration and compared that with replication after IM inoculation. No ASF clinical signs were observed in any of the animals in either group, with animals remaining clinically normal until the day of the challenge, at 28 days post-infection (dpi) (Figure 1). Experimentally, ON inoculation of a high dose of ASFV-G-ΔI177L did not result in the appearance of clinical signs of ASF. 

**Figure 2:**
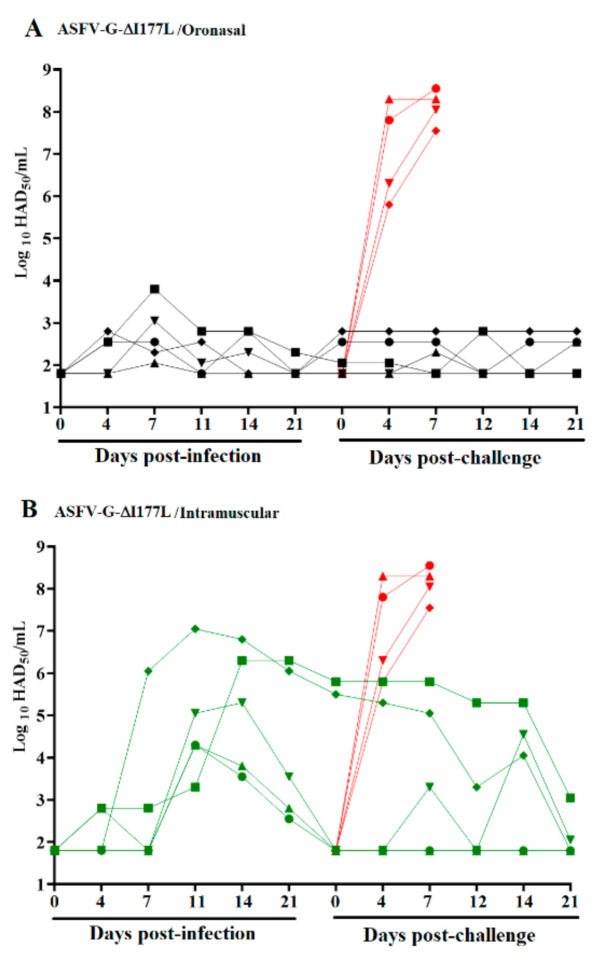
Viremia titers in pigs either oronasally or intramuscularly inoculated with ASFV-G-ΔI177L before and after challenge with ASFV-G (**A**,**B**). Each curve represents individual data from each of the animals under each of the treatments. Red curves represent mock-inoculated animals used as controls during the challenge. The sensitivity of virus detection was ≥1.8 log_10_ HAD_50_/mL.

Viremia, measured by assessing systemic virus replication, indicated that animals with IM ASFV-G-ΔI177L inoculation had high virus titers beginning at 7 dpi, with titers of 10^4^–10^7^ HAD_50_/mL peaking between 11 and 14 dpi (Figure 2). As previously observed [6], viremias in these animals evolved heterogeneously with two animals reaching challenge day (28 dpi) with relatively high titers (approximately 10^3^ HAD_50_/mL) while the other three had undetectable viremias (<10^1.8^ HAD_50_/mL) at the same time point. Conversely, viremias in animals that had an ON ASFV-G-ΔI177L inoculation remained low throughout the experimental period, with peak titers between 10^2^ to 10^4^ HAD_50_/mL at 4–7 dpi, then remaining at <10^3^ HAD_50_/mL throughout the rest of the pre-challenge period with undetectable titers in 4 out 5 animals on challenge day. Based on this data, ON administration of ASFV-G-ΔI177L affected systemic virus replication, since viremias were lower than those measured after IM inoculation, despite the 10,000-fold higher ON inoculation dose. Sentinel animals cohabitating with IM- or ON-inoculated animals remained clinically normal during the 28-day observational period. In addition, no virus was detected in any of the samples from sentinels (all sampled blood time points as well as tonsil and spleen samples obtained at 28 dpi), indicating that transmission of ASFV-G-ΔI177L did not occur among animals even in those inoculated by the ON route. These results indicate that ASFV-G-ΔI177L-inoculated animals, regardless of the route of administration, may not shed enough virus to infect naive pigs during 28 days of cohabitation.

### 3.2. Assessment of Protective Efficacy of ASFV-G-ΔI177L Administered by the ON Route

In previous studies, protection induced by live attenuated viruses was dependent on the ability of the virus to replicate after inoculation [2,3,4,5,6,7,8,14,15,16]. The low viremia titers measured after ON administration of ASFV-G-ΔI177L may correlate with decreased protection after challenge with the parental virulent ASFV-G. Animals that had either an ON or IM inoculation with ASFV-G-ΔI177L were given an IM challenged at 28 days post-infection with 10^2^ HAD_50_ of ASFV-G. An additional group of five naive animals were challenged as a mock-inoculated control group.

**Figure 3:**
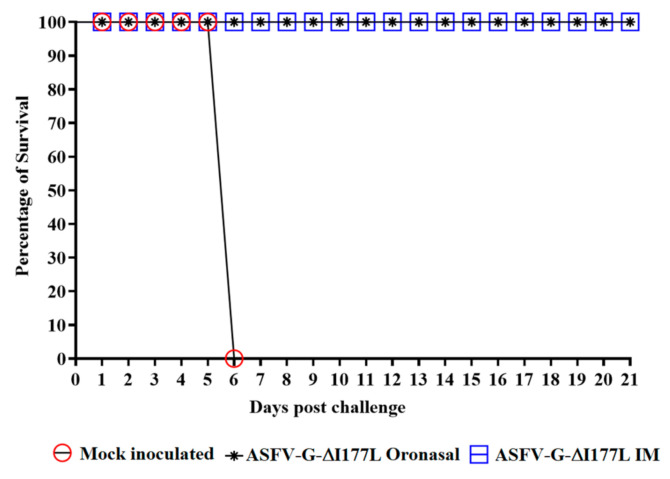
Evolution of mortality in pigs either oronasally or intramuscularly inoculated with ASFV-G-ΔI177L or mock treated and challenged with 10^2^ HAD^50^ of ASFV-G.

As expected, all control animals displayed ASF-related signs beginning at 5 days post-challenge (dpc), with an increased severity in clinical signs until euthanasia at 6 dpc (Table 1 and Figure 3). Animals inoculated with ASFV-G-ΔI177L, regardless of the route of administration, remained clinically normal and showed no signs of disease during the 21-day observational period, thus demonstrating that ASFV-G-ΔI177L offered protection against disease when challenged by the highly virulent parental virus.

Viremia values from mock-inoculated animals infected with ASFV-G were as expected, with high titers (10^5^ to 10^8.5^ HAD_50_/mL) on day 4 pi, increasing (averaging HAD_50_/mL) until day 6 pi, when all animals were euthanized. Conversely, viremia values after challenge in animals intramuscularly inoculated with ASFV-G-ΔI177L progressively decreased in the two animals with high titers at the time of challenge until the end of the experimental period (21 days after challenge), at which time no virus could be detected in one animal, and very low titers (10^3.5^ HAD_50_/mL) were measured in the other (Figure 2). Two of the three animals with undetectable viremia at challenge remained with undetectable titers until the end of the experiment (21 days post challenge - dpc) (Figure 2), while another animal experienced intermittent viremia peaks after challenge with undetectable titers by 21 dpc. After challenge, the group of animals oronasally inoculated with ASFV-G-ΔI177L presented low viremia titers (≤10^2.8^ HAD_50_/mL) throughout the 21-day observational period or had undetectable viremias on the final day of the experiment (Figure 2).

These results indicate that both IM and ON administration of ASFV-G-ΔI177L effectively induces protection against challenge with the virulent parental ASFV-G.

### 3.3. Antibody Response in Animals ON Inoculated with ASFV-G-ΔI177L

**Figure 4:**
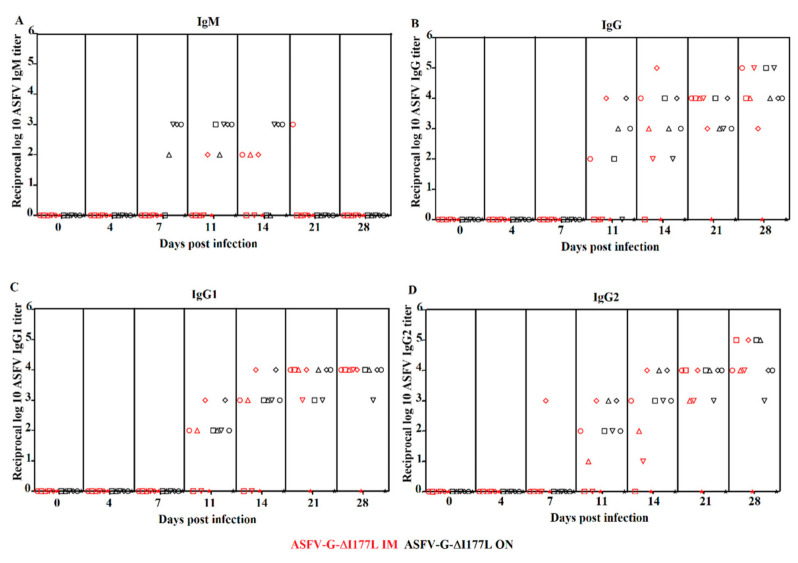
Anti-ASFV antibody titers detected by ELISA in pigs oronasally (ON) or intramuscularly (IM) inoculated with ASFV-G-ΔI177L. Individual serum ASFV-specific antibody titers (indicated by individual symbols) mediated by IgM, total IgG, IgG1 and IgG2 are represented in panels (**A**), (**B**), (**C**) and (**D**), respectively. Titers are expressed as the log_10_ of the reciprocal of the highest dilution of sera at least duplicating OD readings of a pool of mock-infected sera.

Based on results obtained with different attenuated ASFV strains produced in our laboratory, the major immunological parameter consistently associated with protection against disease is the presence of virus-specific circulating antibodies in immunized animals [13]. We evaluated the ability of oronasally administered ASFV-G-ΔI177L to induce a circulating ASFV-specific antibody response and compared that with the response elicited in animals inoculated intramuscularly with ASFV-G-ΔI177L. Serum antibodies were detected using an in-house developed ELISA adapted to quantify antibody response mediated by different immunoglobulin isotypes [6]. The virus-specific IgM-mediated antibody response was detectable at 7 dpi in animals that had an ON inoculation and maintained relatively high titers (10^2^–10^3^) until 14 dpi. The IgM response in IM-inoculated animals appeared delayed and lower compared to that of the ON-inoculated animals (Figure 4A). An IgG-mediated ASFV-specific antibody response was detected by 11 dpi, increasing until 21 dpi. Maximum titers (10^4^–10^5^) were observed by the day of challenge (28 dpi). No differences were observed in the IgG titers of animals inoculated by either the ON or IM route (Figure 4B). Analysis of the virus-specific IgG1- and IgG2-mediated antibody response demonstrated that both isotypes equally contributed to the response, having similar kinetics as that described for IgG (Figure 4C,D). The ASFV-specific antibody response mediated by IgG1 or IgG2 observed in animals inoculated by the ON or the IM route did not differ. Therefore, no major differences were found in the antibody response to ASFV in animals inoculated by either route.

## 4. Discussion

The only efficacious experimental vaccine candidates for the ASFV strain responsible for the current pandemic are live attenuated strains developed by deleting specific virulence-associated genes in the genome of the virulent strain [3,4,5,6,7,15]. Among the different attenuated strains developed in our laboratory [3,4,5,6], the safety and efficacy of ASFV-G-ΔI177L make this experimental vaccine a leading candidate over other live attenuated strains for its commercial potential [6].

An important factor when considering a vaccination program for ASFV is the involvement of wild swine, which has been a constant factor in epidemiological ASF scenarios in both endemic and disease-free areas affected by a disease outbreak [17]. The elimination of infected wild swine constitutes an important component in the control and eradication of ASFV from an affected region. The immunization of wild swine requires oral vaccine administration. To our knowledge, few reports have studied non-parenteral administration of ASF vaccines.

From the sparse reports detailing oral immunization studies, several observations indicate that an oral vaccination is a reasonable approach to protecting wild boar populations against ASF. It has been reported that intranasal (IN) administration of the naturally attenuated strain OURT88/3 provided complete protection against challenge with the virulent homologous isolate OURT88/1, while in this case IM inoculation resulted in less protection [18]. The oral administration of a non-hemadsorbing, naturally attenuated ASFV isolate, Lv17/WB/Rie1, induced protection in wild boars against challenge with the virulent homologous ASF virus isolate Arm07 [19]. A recombinant BeninΔDP148R virus, developed by deleting the DP148R gene from a virulent Benin 97/1 isolate, induced protection against virulent parental virus challenge when immunized by either the IM or the IN route [8]. These studies suggest it is possible to administer ASFV experimental vaccines orally or nasally to induce protection. This effect is likely due to the initial replication of the vaccine virus in the local mucosal entry site, which stimulates the induction of a systemic immune response that protects the animal against subsequent infection. Prior to this study we did not have any previous experience delivering ASFV-G-ΔI177L in the oral or nasal cavity. In this initial study, we decided to administer the vaccine candidate by combining both routes to increase the chances of successful vaccination. 

In this study, we reported that the oronasal administration of ASFV-G-ΔI177L induced protection against challenge by the virulent parental virus ASFV-G and was comparable to that induced by parenteral inoculation of ASFV-G-ΔI177L. Although oronasally administered ASFV-G-ΔI177L replicated less efficiently when parenterally inoculated, it still induced a systemic antibody response that protected the animal against an ASFV-G challenge. These results suggest that the ON administration of ASFV-G-ΔI177L could be a viable delivery method for vaccines. 

## Figures and Tables

**Table 1:** Swine survival and fever response in animals inoculated either intramuscularly or oronasally with ASFV-G-ΔI177L and challenged at 28 dpi with 10^2^ HAD_50_ of virulent ASFV-G.

			Fever		
ASFV-G- (HAD_50_)	No. of Survivors/Total	Mean Time to Death (Days ± SD)	No. of Days to Onset (Days ± SD)	Duration No. of Days (DAYS ± SD)	Maximum Daily Temp °C (± SD)
ΔI177L IM (102)	5/5	-	-	-	39.43 (0.26)
ΔI177L ON (106)	5/5	-	-	-	17.78 (0.4)
Mock	0/5	6 (0) ^(1)^	5.8 (0.45)	1.2 (0.45)	40.67 (0.81)

^(1)^ All animals were euthanized since they all reached the clinical end point as defined in the IACUC protocol.

## Data Availability

Not applicable.
